# Propane-1,2-diammonium bis­(6-carboxy­pyridine-2-carboxyl­ate) dihydrate

**DOI:** 10.1107/S1600536808013263

**Published:** 2008-05-10

**Authors:** Hossein Aghabozorg, Mohammad Heidari, Mohammad Ghadermazi, Jafar Attar Gharamaleki

**Affiliations:** a Faculty of Chemistry, Tarbiat Moallem University, 49 Mofateh Avenue, Tehran, Iran; b Department of Chemistry, Faculty of Science, University of Kurdistan, Sanandaj, Iran

## Abstract

The reaction of propane-1,2-diamine (pn) and pyridine-2,6-dicarboxylic acid (pydcH_2_) in a 1:2 molar ratio in aqueous solution resulted in the formation of the title compound, C_3_H_12_N_2_^2+^·2C_7_H_4_NO_4_·2H_2_O or (pnH_2_)(pydcH)_2_·2H_2_O. The structure contains two monoanionic deprotonated forms of pyridine-2,6-dicarboxylic acid molecules (pydcH)^−^, a diprotonated propane-1,2-diamine (pnH_2_)^2+^, and two water mol­ecules. A significant π–π stacking inter­action is observed between the pyridyl rings of the (pydcH)^−^ fragments, with a face-to-face distance of 3.6194 (9) Å. In the crystal structure, a wide range of non-covalent inter­actions consisting of ion pairing, hydrogen bonding [of the types of O—H⋯O, N—H⋯O, N—H⋯N and C—H⋯O, with *D*⋯*A* distances in the range 2.454 (2)–3.222 (2)Å] and π–π stacking inter­actions [centroid–centroid distance = 3.6194 (9) Å] connect the components into a supra­molecular structure.

## Related literature

For related literature, see: Aghabozorg *et al.* (2007 ▶, 2008 ▶); Aghabozorg, Ghadermazi & Attar Gharamaleki (2006 ▶); Aghabozorg, Ghadermazi & Ramezanipour (2006 ▶).
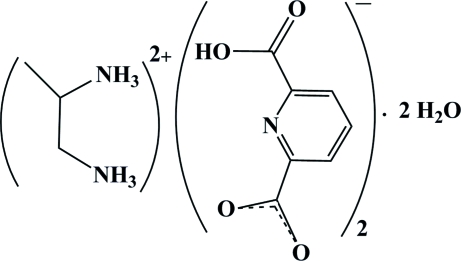


## Experimental

### 

#### Crystal data

C_3_H_12_N_2_^2+^·2C_7_H_4_NO_4_^−^·2H_2_O
*M* *_r_* = 444.40
Triclinic, 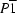

*a* = 7.5587 (3) Å
*b* = 11.0388 (5) Å
*c* = 12.5821 (6) Å
α = 98.533 (1)°
β = 99.844 (1)°
γ = 106.410 (1)°
*V* = 970.52 (7) Å^3^
*Z* = 2
Mo *K*α radiation
μ = 0.13 mm^−1^
*T* = 100 (2) K
0.11 × 0.10 × 0.06 mm

#### Data collection

Bruker SMART APEXII diffractometer
Absorption correction: multi-scan (*SADABS*; Sheldrick, 1996 ▶) *T*_min_ = 0.984, *T*_max_ = 0.993
10335 measured reflections
4242 independent reflections
3220 reflections with *I* > 2σ(*I*)
*R*_int_ = 0.033

#### Refinement

*R*[*F* ^2^ > 2σ(*F* ^2^)] = 0.040
*wR*(*F* ^2^) = 0.104
*S* = 1.04
4242 reflections
280 parameters
H-atom parameters constrained
Δρ_max_ = 0.36 e Å^−3^
Δρ_min_ = −0.34 e Å^−3^

### 

Data collection: *APEX2* (Bruker, 2007 ▶); cell refinement: *APEX2*; data reduction: *APEX2*; program(s) used to solve structure: *SHELXS97* (Sheldrick, 2008 ▶); program(s) used to refine structure: *SHELXL97* (Sheldrick, 2008 ▶); molecular graphics: *SHELXTL* (Sheldrick, 2008 ▶); software used to prepare material for publication: *SHELXL97*.

## Supplementary Material

Crystal structure: contains datablocks I, global. DOI: 10.1107/S1600536808013263/om2231sup1.cif

Structure factors: contains datablocks I. DOI: 10.1107/S1600536808013263/om2231Isup2.hkl

Additional supplementary materials:  crystallographic information; 3D view; checkCIF report

## Figures and Tables

**Table 1:** Hydrogen-bond geometry (Å, °)

*D*—H⋯*A*	*D*—H	H⋯*A*	*D*⋯*A*	*D*—H⋯*A*
O1—H1⋯O8^i^	0.87	1.61	2.479 (2)	175
O1*W*—H1*A*⋯O7^ii^	0.87	1.78	2.649 (2)	177
O1*W*—H1*B*⋯O2*W*^iii^	0.87	1.90	2.751 (2)	166
O2*W*—H2*A*⋯O4^iii^	0.87	2.00	2.855 (2)	169
O2*W*—H2*B*⋯O4	0.87	1.94	2.776 (2)	160
N3—H3*B*⋯N1^iv^	0.91	2.16	2.971 (2)	149
N3—H3*C*⋯O6^v^	0.91	1.92	2.819 (2)	172
N3—H3*D*⋯O1*W*^iii^	0.91	1.88	2.790 (2)	176
N4—H4*B*⋯O1*W*^v^	0.91	1.97	2.854 (2)	163
N4—H4*C*⋯N2^vi^	0.91	2.13	3.017 (2)	166
N4—H4*D*⋯O2^vii^	0.91	2.01	2.884 (2)	160
O5—H5⋯O3^viii^	0.87	1.59	2.454 (2)	178
C16—H16*A*⋯O5^vi^	1.00	2.54	3.182 (2)	122
C16—H16*A*⋯O6^v^	1.00	2.58	3.222 (2)	122

Symmetry codes: (i) 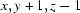; (ii) 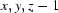; (iii) 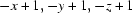; (iv) 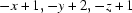; (v) 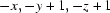; (vi) 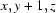; (vii) 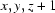; (viii) 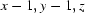.

**Fig. 1.**
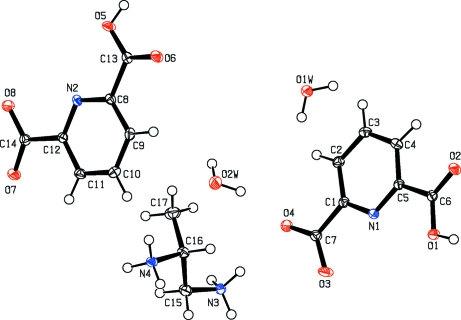
The molecular structure of the title compound, with displacement ellipsoids drawn at the 50% probability level.

**Fig. 2.**
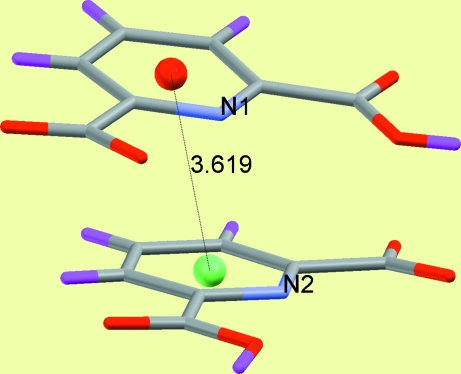
A view of π-π stacking interactions between the two aromatic rings of (pydcH)- fragments with centroid-centroid distance of 3.6194 (9) Å [-x, 1 -y, 1 -z].

**Fig. 3.**
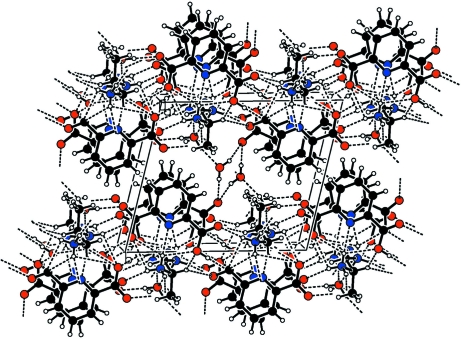
The crystal packing of the title compound with hydrogen bonds shown as dashed lines as viewed approximately down a.

## supplementary crystallographic information

### Comment

Recently, we have defined a plan to prepare water soluble proton-transfer compounds as novel self assembled systems that can function as suitable ligands in the synthesis of metal complexes. In this regard, we have reported cases in which proton transfers from pyridine-2,6-dicarboxylic acid, pydcH_2_, and benzene-1,2,4,5-tetracarboxylic acid, btcH_4_, to propane-1,3-diamine (tn) and 1,10-phenanthroline, (phen). These resulted in the formation of some novel proton transfer compounds such as (pnH_2_)(pydc).(pydcH_2_).2.5H_2_O (Aghabozorg, Ghadermazi, Ramezanipour, 2006), (pnH_2_)_2_(btc).2H_2_O (Aghabozorg, *et al.*, 2007) and (phenH)_4_(btcH_3_)_2_(btcH_2_) (Aghabozorg, Ghadermazi, Attar Gharamaleki, 2006). For more details and related literature see our recent review article (Aghabozorg, *et al.*, 2008).

The molecular structure of the title compound is shown in Fig. 1. The crystal structure shows that a single proton from each of the carboxyl groups was transferred to the propane-1,2-diamine molecule (pn), rendering it a dication. Thus, the negative charges of two monoanionic 6-carboxypyridine-2-carboxylate groups, (pydcH)^-^, are neutralized by a doubly protonated propane-1,2-diammonium, (pnH_2_)^2+^, fragment.

An alternating π-π stacking interaction exits between the two aromatic rings of (pydcH)^-^ with centroid-centroid distance of 3.6194 (9) Å [-*x*, 1 - *y*, 1 - *z*] (Fig. 2).

The C–O distances for this compound support the existence of both ionic and non-ionic acid moieties. The long bond distances of C6–O1 [1.2982 (19) Å] and C13–O5 [1.2936 (19) Å] imply the presence of neutral form of carboxylic acids, whereas the relatively short bond distances of C6–O2 [1.2239 (19) Å] and C13–O6 [1.2260 (19) Å] confirm the presence of double bonds.

A number of O—H···O, N—H···O, N–H···N and C—H···O hydrogen bonds, with D···A distances ranging from 2.454 (2) to 3.222 (2) Å, are observed in the crystal structure of the title compound (Table 1). The shortest hydrogen bond is O5—H5···O3^viii^ (*x* - 1, *y* - 1, *z*) with D···A = 2.454 (2) Å, a strong interaction. Water molecules in this structure increase the number of hydrogen bonding interactions. Ion pairing, π-π stacking and van der Waals interactions are also effective in the packing of the crystal structure. These interactions result in the formation of a supramolecular structure (Fig. 3).

### Experimental

Solutions of propane-1,2-diamine (40 mg, 1 mmol) in THF (10 ml) and pyridine-2,6-dicarboxylic acid (360 mg, 2 mmol) in H_2_O (10 ml) were added to each other in a 1:2 molar ratio, and the reaction mixture was heated at about 40^°^C for 2 h. Yellow crystals of the title compound were obtained from the solution after three weeks at room temperature.

### Refinement

The hydrogen atoms of NH_3_ and OH groups, and also H atoms of water molecules were found in difference Fourier synthesis. The H(C) atom positions were calculated. All H(N) and H(O) atoms were refined in isotropic approximation in rigid model, the H(C) atoms were refined in isotropic approximatiom in riding model with with the *U*_iso_(H) parameters equal to 1.2 *U*_eq_(Xi) for OH, CH and CH_2_ gropus and 1.5 *U*_eq_(Xii) for NH_3_ and CH_3_ group, where U(Xi) and U(Ni) are respectively the equivalent thermal parameters of the atoms to which corresponding H atoms are bonded.

### Crystal data

**Table:**

C_3_H_12_N_2_^2+^·2C_7_H_4_NO_4_^–^·2(H_2_O)	*Z* = 2
*M**_r_* = 444.40	*F*_000_ = 468
Triclinic, *P*1	*D*_x_ = 1.521 Mg m^−^^3^
*a* = 7.5587 (3) Å	Mo *K*α radiation λ = 0.71073 Å
*b* = 11.0388 (5) Å	Cell parameters from 2295 reflections
*c* = 12.5821 (6) Å	θ = 3–27º
α = 98.533 (1)º	µ = 0.13 mm^−^^1^
β = 99.844 (1)º	*T* = 100 (2) K
γ = 106.410 (1)º	Prism, light yellow
*V* = 970.52 (7) Å^3^	0.11 × 0.10 × 0.06 mm

### Data collection

**Table:**

Bruker SMART APEXII diffractometer	4242 independent reflections
Radiation source: fine-focus sealed tube	3220 reflections with *I* > 2σ(*I*)
Monochromator: graphite	*R*_int_ = 0.033
*T* = 100(2) K	θ_max_ = 27.0º
φ and ω scans	θ_min_ = 1.7º
Absorption correction: multi-scan(SADABS; Sheldrick, 1996)	*h* = −9→9
*T*_min_ = 0.984, *T*_max_ = 0.993	*k* = −14→14
10335 measured reflections	*l* = −16→16

### Refinement

**Table:**

Refinement on *F*^2^	Secondary atom site location: difference Fourier map
Least-squares matrix: full	Hydrogen site location: mixed
*R*[*F*^2^ > 2σ(*F*^2^)] = 0.040	H-atom parameters constrained
*wR*(*F*^2^) = 0.104	*w* = 1/[σ^2^(*F*_o_^2^) + (0.05*P*)^2^ + 0.22*P*] where *P* = (*F*_o_^2^ + 2*F*_c_^2^)/3
*S* = 1.04	(Δ/σ)_max_ < 0.001
4242 reflections	Δρ_max_ = 0.36 e Å^−^^3^
280 parameters	Δρ_min_ = −0.34 e Å^−^^3^
Primary atom site location: structure-invariant direct methods	Extinction correction: none

### Special details

**Table:**

Geometry. All e.s.d.'s (except the e.s.d. in the dihedral angle between two l.s. planes) are estimated using the full covariance matrix. The cell e.s.d.'s are taken into account individually in the estimation of e.s.d.'s in distances, angles and torsion angles; correlations between e.s.d.'s in cell parameters are only used when they are defined by crystal symmetry. An approximate (isotropic) treatment of cell e.s.d.'s is used for estimating e.s.d.'s involving l.s. planes.
Refinement. Refinement of *F*^2^ against ALL reflections. The weighted *R*-factor *wR* and goodness of fit *S* are based on *F*^2^, conventional *R*-factors *R* are based on *F*, with *F* set to zero for negative *F*^2^. The threshold expression of *F*^2^ > σ(*F*^2^) is used only for calculating *R*-factors(gt) *etc*. and is not relevant to the choice of reflections for refinement. *R*-factors based on *F*^2^ are statistically about twice as large as those based on *F*, and *R*- factors based on ALL data will be even larger.

### Fractional atomic coordinates and isotropic or equivalent isotropic displacement parameters (Å^2^)

**Table:**

	*x*	*y*	*z*	*U*_iso_*/*U*_eq_	
O1	0.30330 (16)	0.96417 (11)	0.10525 (9)	0.0158 (3)	
H1	0.2728	1.0122	0.0610	0.019*	
O2	0.12513 (17)	0.79720 (11)	−0.03137 (9)	0.0171 (3)	
O3	0.62921 (18)	0.89335 (11)	0.43063 (10)	0.0234 (3)	
O4	0.58090 (17)	0.69734 (11)	0.46855 (9)	0.0176 (3)	
N1	0.38792 (19)	0.80055 (12)	0.23258 (11)	0.0117 (3)	
C1	0.4249 (2)	0.71578 (15)	0.29333 (13)	0.0120 (3)	
C2	0.3517 (2)	0.58307 (15)	0.25576 (13)	0.0139 (3)	
H2C	0.3769	0.5267	0.3023	0.017*	
C3	0.2419 (2)	0.53364 (16)	0.14993 (14)	0.0150 (3)	
H3A	0.1918	0.4431	0.1220	0.018*	
C4	0.2069 (2)	0.61941 (16)	0.08566 (14)	0.0145 (3)	
H4A	0.1350	0.5888	0.0118	0.017*	
C5	0.2784 (2)	0.75089 (15)	0.13067 (13)	0.0117 (3)	
C6	0.2292 (2)	0.84218 (15)	0.06044 (13)	0.0126 (3)	
C7	0.5543 (2)	0.77185 (15)	0.40738 (14)	0.0139 (3)	
O5	−0.17333 (17)	0.01198 (11)	0.61266 (10)	0.0175 (3)	
H5	−0.2412	−0.0312	0.5480	0.021*	
O6	−0.18863 (17)	0.17699 (11)	0.53087 (9)	0.0180 (3)	
O7	0.41198 (17)	0.28482 (12)	1.04946 (10)	0.0210 (3)	
O8	0.20387 (17)	0.08880 (11)	0.97111 (9)	0.0171 (3)	
N2	0.08176 (19)	0.17610 (13)	0.79041 (11)	0.0114 (3)	
C8	0.0191 (2)	0.22487 (15)	0.70500 (13)	0.0121 (3)	
C9	0.0797 (2)	0.35514 (16)	0.70194 (13)	0.0138 (3)	
H9A	0.0317	0.3853	0.6397	0.017*	
C10	0.2115 (2)	0.44020 (16)	0.79162 (14)	0.0154 (4)	
H10A	0.2559	0.5299	0.7923	0.019*	
C11	0.2769 (2)	0.39153 (16)	0.87986 (13)	0.0139 (3)	
H11A	0.3670	0.4478	0.9425	0.017*	
C12	0.2104 (2)	0.26003 (15)	0.87667 (13)	0.0111 (3)	
C13	−0.1263 (2)	0.13271 (15)	0.60740 (13)	0.0130 (3)	
C14	0.2828 (2)	0.20755 (15)	0.97451 (13)	0.0132 (3)	
N3	0.41675 (19)	0.91821 (13)	0.68441 (11)	0.0129 (3)	
H3B	0.5070	0.9960	0.6940	0.019*	
H3C	0.3499	0.8938	0.6132	0.019*	
H3D	0.4730	0.8583	0.7004	0.019*	
C15	0.2867 (2)	0.92892 (17)	0.75906 (14)	0.0180 (4)	
H15A	0.2953	1.0207	0.7806	0.022*	
H15B	0.3282	0.8996	0.8269	0.022*	
C16	0.0823 (2)	0.85019 (15)	0.70690 (14)	0.0145 (3)	
H16A	0.0451	0.8752	0.6352	0.017*	
C17	0.0413 (3)	0.70574 (17)	0.68557 (16)	0.0230 (4)	
H17A	−0.0931	0.6631	0.6510	0.034*	
H17B	0.0719	0.6791	0.7555	0.034*	
H17C	0.1184	0.6812	0.6363	0.034*	
N4	−0.03197 (19)	0.88970 (13)	0.78355 (11)	0.0130 (3)	
H4B	−0.1564	0.8435	0.7566	0.019*	
H4C	−0.0168	0.9754	0.7894	0.019*	
H4D	0.0078	0.8743	0.8512	0.019*	
O1W	0.41860 (16)	0.26610 (11)	0.25748 (9)	0.0162 (3)	
H1B	0.4897	0.3358	0.3049	0.019*	
H1A	0.4198	0.2719	0.1894	0.019*	
O2W	0.33952 (17)	0.54108 (12)	0.57226 (10)	0.0204 (3)	
H2B	0.3933	0.5814	0.5260	0.024*	
H2A	0.3485	0.4636	0.5598	0.024*	

### Atomic displacement parameters (Å^2^)

**Table:**

	*U*^11^	*U*^22^	*U*^33^	*U*^12^	*U*^13^	*U*^23^
O1	0.0200 (6)	0.0109 (6)	0.0138 (6)	0.0032 (5)	−0.0022 (5)	0.0045 (5)
O2	0.0199 (6)	0.0167 (6)	0.0112 (6)	0.0044 (5)	−0.0032 (5)	0.0026 (5)
O3	0.0325 (8)	0.0109 (6)	0.0166 (6)	0.0008 (5)	−0.0103 (6)	0.0018 (5)
O4	0.0226 (7)	0.0144 (6)	0.0135 (6)	0.0041 (5)	−0.0012 (5)	0.0055 (5)
N1	0.0114 (7)	0.0118 (7)	0.0106 (6)	0.0026 (5)	0.0014 (5)	0.0020 (5)
C1	0.0105 (8)	0.0130 (8)	0.0126 (8)	0.0033 (6)	0.0029 (6)	0.0030 (6)
C2	0.0152 (8)	0.0120 (8)	0.0155 (8)	0.0053 (6)	0.0027 (7)	0.0049 (6)
C3	0.0156 (8)	0.0108 (8)	0.0170 (8)	0.0032 (6)	0.0030 (7)	0.0005 (6)
C4	0.0133 (8)	0.0151 (8)	0.0125 (8)	0.0040 (7)	0.0001 (6)	−0.0002 (6)
C5	0.0092 (7)	0.0131 (8)	0.0125 (8)	0.0029 (6)	0.0024 (6)	0.0028 (6)
C6	0.0102 (8)	0.0153 (8)	0.0120 (8)	0.0030 (6)	0.0030 (6)	0.0029 (6)
C7	0.0156 (8)	0.0120 (8)	0.0139 (8)	0.0047 (6)	0.0024 (7)	0.0025 (6)
O5	0.0208 (6)	0.0129 (6)	0.0130 (6)	0.0027 (5)	−0.0052 (5)	0.0006 (5)
O6	0.0221 (7)	0.0159 (6)	0.0121 (6)	0.0045 (5)	−0.0040 (5)	0.0030 (5)
O7	0.0225 (7)	0.0188 (7)	0.0135 (6)	−0.0003 (5)	−0.0056 (5)	0.0032 (5)
O8	0.0211 (6)	0.0132 (6)	0.0144 (6)	0.0040 (5)	−0.0017 (5)	0.0042 (5)
N2	0.0108 (7)	0.0140 (7)	0.0094 (6)	0.0042 (5)	0.0017 (5)	0.0020 (5)
C8	0.0110 (8)	0.0145 (8)	0.0106 (8)	0.0045 (6)	0.0023 (6)	0.0015 (6)
C9	0.0138 (8)	0.0164 (9)	0.0128 (8)	0.0052 (7)	0.0035 (6)	0.0058 (6)
C10	0.0156 (8)	0.0123 (8)	0.0184 (9)	0.0035 (7)	0.0041 (7)	0.0046 (6)
C11	0.0111 (8)	0.0142 (8)	0.0133 (8)	0.0016 (6)	0.0011 (6)	−0.0001 (6)
C12	0.0096 (7)	0.0125 (8)	0.0110 (8)	0.0031 (6)	0.0033 (6)	0.0014 (6)
C13	0.0137 (8)	0.0140 (8)	0.0118 (8)	0.0053 (6)	0.0027 (6)	0.0027 (6)
C14	0.0144 (8)	0.0132 (8)	0.0128 (8)	0.0054 (7)	0.0035 (7)	0.0024 (6)
N3	0.0131 (7)	0.0123 (7)	0.0127 (7)	0.0035 (5)	0.0014 (6)	0.0032 (5)
C15	0.0154 (9)	0.0192 (9)	0.0162 (8)	0.0026 (7)	0.0038 (7)	−0.0004 (7)
C16	0.0136 (8)	0.0149 (8)	0.0144 (8)	0.0044 (7)	0.0029 (7)	0.0013 (6)
C17	0.0234 (10)	0.0160 (9)	0.0278 (10)	0.0036 (7)	0.0090 (8)	0.0007 (7)
N4	0.0129 (7)	0.0123 (7)	0.0121 (7)	0.0033 (5)	0.0003 (5)	0.0019 (5)
O1W	0.0175 (6)	0.0152 (6)	0.0134 (6)	0.0030 (5)	0.0006 (5)	0.0027 (5)
O2W	0.0261 (7)	0.0168 (6)	0.0190 (6)	0.0065 (5)	0.0062 (5)	0.0051 (5)

### Geometric parameters (Å, °)

**Table:**

O1—C6	1.2982 (19)	C9—H9A	0.9500
O1—H1	0.8700	C10—C11	1.380 (2)
O2—C6	1.2239 (19)	C10—H10A	0.9500
O3—C7	1.266 (2)	C11—C12	1.387 (2)
O4—C7	1.240 (2)	C11—H11A	0.9500
N1—C5	1.343 (2)	C12—C14	1.520 (2)
N1—C1	1.351 (2)	N3—C15	1.487 (2)
C1—C2	1.387 (2)	N3—H3B	0.9100
C1—C7	1.521 (2)	N3—H3C	0.9100
C2—C3	1.382 (2)	N3—H3D	0.9100
C2—H2C	0.9500	C15—C16	1.516 (2)
C3—C4	1.384 (2)	C15—H15A	0.9900
C3—H3A	0.9500	C15—H15B	0.9900
C4—C5	1.388 (2)	C16—N4	1.496 (2)
C4—H4A	0.9500	C16—C17	1.509 (2)
C5—C6	1.516 (2)	C16—H16A	1.0000
O5—C13	1.2936 (19)	C17—H17A	0.9800
O5—H5	0.8701	C17—H17B	0.9800
O6—C13	1.2260 (19)	C17—H17C	0.9800
O7—C14	1.244 (2)	N4—H4B	0.9100
O8—C14	1.2684 (19)	N4—H4C	0.9100
N2—C8	1.346 (2)	N4—H4D	0.9100
N2—C12	1.347 (2)	O1W—H1B	0.8700
C8—C9	1.389 (2)	O1W—H1A	0.8699
C8—C13	1.512 (2)	O2W—H2B	0.8700
C9—C10	1.386 (2)	O2W—H2A	0.8700
			
C6—O1—H1	111.7	C11—C12—C14	119.22 (14)
C5—N1—C1	116.90 (13)	O6—C13—O5	125.65 (15)
N1—C1—C2	122.99 (15)	O6—C13—C8	118.45 (14)
N1—C1—C7	117.01 (14)	O5—C13—C8	115.90 (14)
C2—C1—C7	119.99 (14)	O7—C14—O8	126.73 (15)
C3—C2—C1	119.31 (15)	O7—C14—C12	117.09 (14)
C3—C2—H2C	120.3	O8—C14—C12	116.18 (14)
C1—C2—H2C	120.3	C15—N3—H3B	109.5
C2—C3—C4	118.32 (15)	C15—N3—H3C	109.5
C2—C3—H3A	120.8	H3B—N3—H3C	109.5
C4—C3—H3A	120.8	C15—N3—H3D	109.5
C3—C4—C5	119.02 (15)	H3B—N3—H3D	109.5
C3—C4—H4A	120.5	H3C—N3—H3D	109.5
C5—C4—H4A	120.5	N3—C15—C16	112.99 (14)
N1—C5—C4	123.36 (15)	N3—C15—H15A	109.0
N1—C5—C6	118.79 (14)	C16—C15—H15A	109.0
C4—C5—C6	117.85 (14)	N3—C15—H15B	109.0
O2—C6—O1	125.56 (15)	C16—C15—H15B	109.0
O2—C6—C5	119.01 (14)	H15A—C15—H15B	107.8
O1—C6—C5	115.43 (13)	N4—C16—C17	110.23 (14)
O4—C7—O3	125.45 (15)	N4—C16—C15	106.01 (13)
O4—C7—C1	118.87 (14)	C17—C16—C15	115.19 (15)
O3—C7—C1	115.66 (14)	N4—C16—H16A	108.4
C13—O5—H5	106.7	C17—C16—H16A	108.4
C8—N2—C12	117.06 (14)	C15—C16—H16A	108.4
N2—C8—C9	123.62 (15)	C16—C17—H17A	109.5
N2—C8—C13	118.08 (14)	C16—C17—H17B	109.5
C9—C8—C13	118.30 (14)	H17A—C17—H17B	109.5
C10—C9—C8	118.53 (15)	C16—C17—H17C	109.5
C10—C9—H9A	120.7	H17A—C17—H17C	109.5
C8—C9—H9A	120.7	H17B—C17—H17C	109.5
C11—C10—C9	118.47 (15)	C16—N4—H4B	109.5
C11—C10—H10A	120.8	C16—N4—H4C	109.5
C9—C10—H10A	120.8	H4B—N4—H4C	109.5
C10—C11—C12	119.66 (15)	C16—N4—H4D	109.5
C10—C11—H11A	120.2	H4B—N4—H4D	109.5
C12—C11—H11A	120.2	H4C—N4—H4D	109.5
N2—C12—C11	122.65 (15)	H1B—O1W—H1A	113.3
N2—C12—C14	118.13 (14)	H2B—O2W—H2A	106.8
			
C5—N1—C1—C2	−1.5 (2)	C12—N2—C8—C13	−179.63 (14)
C5—N1—C1—C7	178.02 (14)	N2—C8—C9—C10	−0.2 (2)
N1—C1—C2—C3	2.7 (3)	C13—C8—C9—C10	179.40 (15)
C7—C1—C2—C3	−176.84 (15)	C8—C9—C10—C11	0.1 (2)
C1—C2—C3—C4	−0.9 (2)	C9—C10—C11—C12	0.2 (2)
C2—C3—C4—C5	−1.8 (2)	C8—N2—C12—C11	0.4 (2)
C1—N1—C5—C4	−1.4 (2)	C8—N2—C12—C14	179.70 (14)
C1—N1—C5—C6	178.54 (14)	C10—C11—C12—N2	−0.5 (2)
C3—C4—C5—N1	3.1 (3)	C10—C11—C12—C14	−179.77 (15)
C3—C4—C5—C6	−176.86 (15)	N2—C8—C13—O6	177.64 (15)
N1—C5—C6—O2	−178.03 (15)	C9—C8—C13—O6	−2.0 (2)
C4—C5—C6—O2	1.9 (2)	N2—C8—C13—O5	−2.6 (2)
N1—C5—C6—O1	1.7 (2)	C9—C8—C13—O5	177.79 (14)
C4—C5—C6—O1	−178.34 (14)	N2—C12—C14—O7	174.56 (15)
N1—C1—C7—O4	175.02 (15)	C11—C12—C14—O7	−6.1 (2)
C2—C1—C7—O4	−5.4 (2)	N2—C12—C14—O8	−5.7 (2)
N1—C1—C7—O3	−6.2 (2)	C11—C12—C14—O8	173.67 (15)
C2—C1—C7—O3	173.32 (15)	N3—C15—C16—N4	−170.15 (13)
C12—N2—C8—C9	−0.1 (2)	N3—C15—C16—C17	67.7 (2)

### Hydrogen-bond geometry (Å, °)

**Table:**

*D*—H···*A*	*D*—H	H···*A*	*D*···*A*	*D*—H···*A*
O1—H1···O8^i^	0.87	1.61	2.479 (2)	175
O1W—H1A···O7^ii^	0.87	1.78	2.649 (2)	177
O1W—H1B···O2W^iii^	0.87	1.90	2.751 (2)	166
O2W—H2A···O4^iii^	0.87	2.00	2.855 (2)	169
O2W—H2B···O4	0.87	1.94	2.776 (2)	160
N3—H3B···N1^iv^	0.91	2.16	2.971 (2)	149
N3—H3C···O6^v^	0.91	1.92	2.819 (2)	172
N3—H3D···O1W^iii^	0.91	1.88	2.790 (2)	176
N4—H4B···O1W^v^	0.91	1.97	2.854 (2)	163
N4—H4C···N2^vi^	0.91	2.13	3.017 (2)	166
N4—H4D···O2^vii^	0.91	2.01	2.884 (2)	160
O5—H5···O3^viii^	0.87	1.59	2.454 (2)	178
C16—H16A···O5^vi^	1.00	2.54	3.182 (2)	122
C16—H16A···O6^v^	1.00	2.58	3.222 (2)	122

Symmetry codes: (i) *x*, *y*+1, *z*−1; (ii) *x*, *y*, *z*−1; (iii) −*x*+1, −*y*+1, −*z*+1;  (iv) −*x*+1, −*y*+2, −*z*+1; (v) −*x*, −*y*+1, −*z*+1; (vi) *x*, *y*+1, *z*; (vii) *x*, *y*, *z*+1; (viii) *x*−1, *y*−1, *z*.
